# Evaluation of the Capillary Blood Glucose Self-monitoring Program

**DOI:** 10.1590/0104-1169.3282.2483

**Published:** 2014

**Authors:** Mariana Cristina Augusto, Maria José Trevizani Nitsche, Cristina Maria Garcia de Lima Parada, Maria Lúcia Zanetti, Maria Antonieta de Barros Leite Carvalhaes

**Affiliations:** 1 MSc, RN, Prefeitura Municipal de Botucatu, Botucatu, SP, Brazil; 2 PhD, Professor, Departamento de Enfermagem, Faculdade de Medicina de Botucatu, Universidade Estadual Paulista "Júlio de Mesquita Filho", Botucatu, SP, Brazil; 3 PhD, Adjunct Professor, Departamento de Enfermagem, Faculdade de Medicina de Botucatu, Universidade Estadual Paulista "Júlio de Mesquita Filho" , Botucatu, SP, Brazil; 4 PhD, Associate Professor, Escola de Enfermagem de Ribeirão Preto, Universidade de São Paulo, WHO Collaborating Centre for Nursing Research Development, Ribeirão Preto, SP, Brazil

**Keywords:** Health Evaluation, Diabetes Mellitus, Program Evaluation

## Abstract

**OBJECTIVE:**

to evaluate the structure, process and results of the Capillary Blood Glucose Self-monitoring Program in a Brazilian city.

**METHOD:**

epidemiological, cross-sectional study. The methodological framework of Donabedian was used to construct indicators of structure, process and outcome. A random sample (n = 288) of users enrolled and 96 health professionals who worked in the program was studied. Two questionnaires were used that were constructed for this study, one for professionals and one for users, both containing data for the evaluation of structure, process and outcome. Anthropometric measures and laboratory results were collected by consulting the patients' health records. The analysis involved descriptive statistics.

**RESULTS:**

most of the professionals were not qualified to work in the program and were not knowledgeable about the set of criteria for patient registration. None of the patients received complete and correct orientations about the program and the percentage with skills to perform conducts autonomously was 10%. As regards the result indicators, 86.4% of the patients and 81.3% of the professionals evaluated the program positively.

**CONCLUSION:**

the evaluation indicators designed revealed that one of the main objectives of the program, self-care skills, has not been achieved.

## Introduction

The assessment of health services, technologies and programs has been greatly stimulated in Brazil, for monitoring health professionals' performance as well as for managers to make decisions about human resources and inputs in health, at the federal, state and municipal level.

The National Performance Assessment Policy of the Unified Health System (SUS) indicates that health assessment permits the adoption of intervention measures in response to possible distortions, contradictions and difficulties met in the health services^(^ 1 ^)^. Nevertheless, there are difficulties to established institutional evaluation processes in Brazil, due to the lack of tradition and understanding about the need for the professionals themselves to assess the service they are inserted in^(^ 1 ^-^ 2 ^)^. In fact, the assessment of health services is a complex process that involves political, social, cultural, educational and financial aspects^(^ 3 ^)^.

Among the ongoing health programs in Brazil, the Distribution Program of Glucometers and Inputs for Self-Monitoring of Capillary Glucose (PAMGC) for Diabetes Mellitus (DM) patients is highlighted. The PAMGC was implemented after the approval of Brazilian Federal Law 11.347 in 2006, making obligatory the supply of materials free of charge, such as glucometers, reactive strips for capillary glucose measures and digital puncture lancets, to type 1 (DM 1) and type 2 (DM 2) Diabetes Mellitus patients on insulin therapy for the purpose of self-monitoring at home^(^ 4 ^)^.

Capillary glucose self-monitoring at home allows DM patients to develop skills with a view to autonomy and decision-making to achieve good glucose control measures, reduce acute and chronic complications and, consequently, improve their quality of life. This care technology is recommended as an essential part of the therapeutic strategies for adequate control of DM 1^(^ 5 ^-^ 6 ^)^. Recently, its efficacy in DM 2 patients on insulin therapy has also been proven^(^ 7 ^-^ 8 ^)^, when the capillary glucose measures are used for treatment adjustments. In Brazil, a clinical trial involving DM 1 patients evidenced improvements in metabolic control as a result of capillary glucose self-monitoring^(^ 9 ^)^ .

Nevertheless, Brazilian literature about the assessment of the PAMGC is scarce^(10) ^and the effectiveness of this Program and its benefits for the control of the disease are unknown. Thus, the objective in this study was to assess the structure, process and outcome of the Capillary Glucose Self-Monitoring Program in course in a medium-sized city in the interior of the State of São Paulo, Brazil. The intent is to offer support for the reorientation of the PAMGC proposed by the Ministry of Health, so as to improve the metabolic control of health service users.

## Method

In this epidemiological and cross-sectional study, the PAMGC was assessed in a medium-sized city in the interior of the State of São Paulo in 2010. Therefore, Donabedian's methodological reference framework was used^(^ 2 ^-^ 3 ^)^ to construct the quality indicators of three components: structure, process and outcome.

In the structure assessment, the investigation of the training of the health professionals and patients enrolled in the PAMGC at the start of its implementation was privileged. In the process context, the intent was to assess whether the activities/actions the health professionals referred were those recommended by the technical and standards of the PAMGC and the frequency of the health service users self-monitoring. As regards the outcome component, the proportion of health service users with self-care skills was selected as the main indicator, that is, who were capable of: analyzing the glucose levels, because they are familiar with the normal parameters; recognizing signs and symptoms of hyper and hypoglycemia and using these data for decision making, according to their knowledge level about the disease. In addition, the health service professionals and users' subjective perception was assessed about the benefits deriving from the Program as an outcome indicator.

The normative and technical frameworks used were: Federal Law 11.347 (2006), which established the compulsory supply of glucometers and inputs by the SUS^(^ 4 ^)^; Decree 2.583 from 2007, which regulates how the inputs should be made available^(^ 11 ^)^; the recommendations for care delivery to DM patients on insulin by the Ministry of Health^(^ 12 ^)^ and the Brazilian Diabetes Society^(^ 6 ^)^. The city adopts these frameworks to direct care delivery to DM patients in the public primary healthcare network.

The eligible population consisted of 1,132 individuals over 18 years of age registered in the PAMGC of the city under study between 2006 and June 2009. Thus, it was guaranteed that all individuals for inclusion in the sample would be enrolled in the Program for at least one year before the start of the data collection.

The sample included 288 DM 1 or DM 2 patients on insulin, with a 5% error and 95% reliability coefficient, in view of an unknown prevalence (50%) and study power corresponding to 80%. 

The users were numbered at each health unit and randomly selected through a draft performed in statistical software. Considering possible losses due to deaths, incorrect addresses and refusals, the researchers decided to systematically replace any previously drafted user who could not be contacted by the immediately subsequent user.

To achieve the sample size (288), 326 individuals were drafted and contacted, making the replacements needed due to losses (ten deaths, 22 refusals to participate and six users who were not located). 

As regards the professionals, no draft was used for sampling purposes. Instead, all of them were considered eligible for the study. Ninety-six out of 106 secondary and higher education professionals were investigated who worked with the health service users registered in the Program, including 49 nurse's aides and seven nursing technicians, 19 baccalaureate nurses and 21 physicians (general practitioners and general clinicians). The losses (N=10) were due to refusals and/or impossibility to contact the user after three attempts.

**Figure 1:**
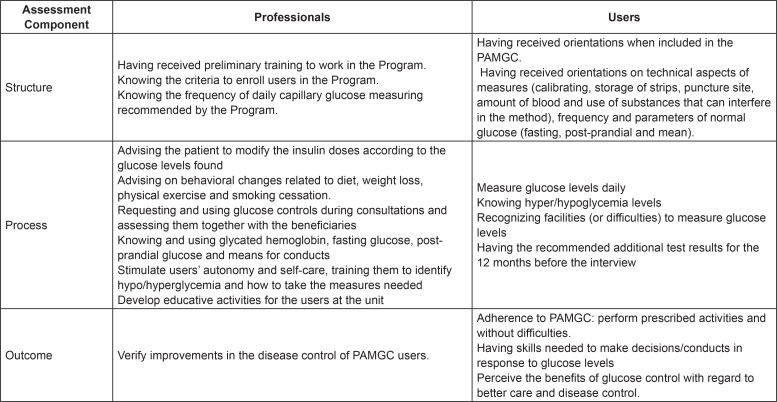
- Structure, process and outcome variables used in PAMGC assessment, Botucatu, 2010.

To collect the data, two instruments were elaborated, involving professionals specialized in assessment, DM and nutrition, given the lack of earlier studies that assessed the PAMGC. The questionnaires went through a pretest, reformulation and pilot test, registering any doubts for the purpose of correction and further application to the selected sample. The first questionnaire was aimed at collecting data about the sociodemographic and clinical variables of the patients enrolled in the Program (gender, age, skin color, marital status, education, diabetes type, time since diagnosis of the disease, length of insulin use and date of inclusion in the Program). The second questionnaire was used to collect the variables related to the structure, process and outcome assessment, presented in Figure 1.

In addition, the patient files were consulted in the Primary Healthcare services where they were enrolled, so as to obtain test results related to the most recent consultation, such as: urea, creatinine, blood culture, simple urine, cholesterol and fractions, fasting glucose and glycated hemoglobin.

The interviews were scheduled and held at the Primary Healthcare services (83% of cases) by four interviewers, two nurses and two undergraduate nursing students, who had been trained and were supervised by one of the authors. For 17% of the users, the interview was held at home due to reduced mobility. The health professionals were interviewed at their units, upon appointment. All interviews were held in June and July 2010.

The data were typed in an Excel worksheet, version 2007, verified and, after consistency analysis and correction of possible errors, transferred for analysis in SPSS 12.0. The individuals' absolute and relative frequencies were calculated for each variable and in combination, and the indicators were estimated.

Approval for the project was obtained from the Research Ethics Committee at Botucatu Medical School, UNESP, protocol 338/2009. To guarantee the professionals' anonymity and further their participation in the study, no data were collected about their characteristics, such as age, gender and experience in the city's primary healthcare network. In addition, measures were taken for the interviews to take place under conditions that guaranteed the interviewees' privacy. 

## Results

The patients studied were predominantly women (63.9%), white (88.2%), married (57.3%), over 60 (63.6%) and with up to four years of education (63.9%). As for how long ago they had been diagnosed with DM, 59.7% had known their condition for more than 11 years and 95.1% suffered from DM 2. Half (50.7%) of the patients had been on insulin for more than six years and 54.9% were registered in the PAMGC in the first semester of 2009.

As regards the knowledge about the criteria for registration in the Program, only 9.4% of the health professionals had received training to work in the Program and only four (4.2%) knew the frequency of daily capillary glucose verification (Table 1).

**Table 1:** - Structure indicators for the health professionals working in the Distribution Program of Glucometers and Inputs for Self-Monitoring of Capillary Glucose, Botucatu, SP, Brazil, 2010.

Structure indicators		Profissionals (N=96)											
		Physicians			Nurses			Secondary level			Total		p*
		N	%		N	%		N	%		N	%	
Preliminary training													0,717
	Yes	1	4,8		2	10,5		6	10,7		9	9,4	
	No	20	95,2		17	89,5		50	89,3		87	90,6	
Knowledge of registration criteria													0,719
	All criteria	-	-		-	-		-	-		-	0	
	Only one criterion	17	81		17	89,5		46	82,1		80	83,3	
	Does not know criteria	4	19		2	10,5		10	17,9		16	16,7	
Frequency of daily capillary glucose verification													<0,0001
	Yes	4	19		-	-		-	-		4	4,2	
	No	17	81		19	100		56	100		92	95,8	

* P-value, chi-square test

As regards the information needed to start their participation in the Program, 11 (3.8%) patients indicated they received information about self-monitoring and only nine (3.1%) referred to the glucose levels that correctly indicate normality, hyper or hypoglycemia (Table 2).

**Table 2:** - Structure indicators for patients registered in the Distribution Program of Glucometers and Inputs for Self- Monitoring of Capillary Glucose, Botucatu, SP, Brazil, 2010.

Structure indicators		Pacients	
		N	%
Orientations when registering in PAMGC			
	Yes	253	87,8
	No	35	12,2
Orientations about technical aspects of measuring			
	Calibration. storage of strips. puncture site. amount of blood. use of substances that can interfere in the method	11	3,8
	Received between two and four correct orientations	50	17,4
	Received one correct orientation	144	50,0
	Received incorrect orientations or does not recall the orientations	83	28,8
Orientations about frequency of capillary glucose verifications			
	Yes	247	85,8
	No	41	14,2
Orientations about frequency of capillary glucose verifications			
	Follow Ministry of Health recommendations	231	80,2
	Differ from Ministry of Health indications or did not receive orientations	57	19,8
Orientations about glucose control parameters			
	Yes	186	65,6
	No	102	35,4
Orientations about capillary glucose parameters			
	Know correct fasting. mean and post-prandial glucose levels	9	3,1
	Know correct values of one or two glucose control parameters	154	53,5
	Indicate incorrect values for two or more parameters	23	8,0
	Patient did not receive orientation	102	35,4

### Process Assessment

Regarding the health professionals, most physicians indicated that they do not advise the patients to adjust the insulin doses according to the capillary glucose results obtained at home. Forty-five percent of the professionals regularly request the recommended laboratory tests to monitor DM patients on insulin, with nurses demonstrating greater compliance with official protocols. The proportions of professionals who referred using and who correctly indicated the normality parameters of fasting (39.6%), and post-prandial capillary glucose (13.5%) and glycated hemoglobin (HbA1c) (12.5%) were low. (Table 3).

**Table 3:** - Process indicators for professionals working in the Distribution Program of Glucometers and Inputs for Self- Monitoring of Capillary Glucose, Botucatu, SP, Brazil, 2010.

Process indicators		Profissionals								
		Physicians (N=21)		Nurses (N=19)		Auxiliaries and technicians (N=56)		Total (N=96)		P
		N	%	N	%	N	%	N	%	
Advises the patient to modify insulin doses (N=21)										
	Yes	6	28,6							
	No	15	71,4							0,421
Diet, weight loss and physical exercise										
	Diet, weight loss, physical exercise and smoking cessation	2	9,5	2	10,5	1	1,7	5	5,2	
	Diet, weight loss and physical exercise	13	61,9	10	52,6	28	50,0	51	53,1	
	Diet and weight loss	4	19,0	6	31,6	23	41,1	33	34,4	
	No lifestyle orientation	2	9,5	1	5,30	4	7,2	7	7,3	
Requests and verifies glucose controls during consultations (N=40)										
	Yes	21	100	19	100					
	No	-	-	-	-					0,034
Requests recommended tests (N=40)										
	All (fasting glucose, lipid profile, glycated hemoglobin, urea and creatinine)	6	28,6	12	63,1			18	45,0	
	Requests at least glycated hemoglobin and fasting glucose	15	71,4	6	31,6			21	52,5	
	Does not request minimal tests (glycated hemoglobin and fasting glucose)	-		1	5,3			1	2,5	0,016
Knows levels and uses glycated hemoglobin for conducts (N=40)										
	Yes	4	19,1	1	5,2			5	12,5	
	No	17	80,9	18	94,7			35	87,5	0,647
Knows levels and uses fasting glucose for conducts (N=96)										
	Yes	8	38,1	8	42,1	22	39,3	38	39,6	
	No	13	61,9	11	57,9	34	60,7	58	60,4	
Knows correct values and uses post-prandial glucose (N=96)										0,671
	Yes	3	14,3	4	21,1	6	10,7	13	13,5	
	No	18	85,7	15	78,9	50	89,3	83	86,5	
Knows correct values and uses mean glucose (N=96)										0,056
	Yes	4	19,1	3	15,8	2	3,6	9	9,4	
	No	17	80,9	16	84,2	54	96,4	87	90,6	

Differences were found between the information referred by the professionals and that found in the health files with regard to the laboratory tests. Most of the health professionals (97.5%) indicated that they request at least one fasting glucose and HbA1C test for the health service users; 67% of them had no records of these tests in their files for the twelve months before the data collection. 

Another process indicator highlighted is that 90.3% of the health service users indicated that they manipulate the glucometer and measure their glucose easily, although the Health services do not offer regular educative activities to train the patients for adherence to the monitoring process (data not shown in Table).

In Table 4, the results of the Program's structure, process and outcome assessment are summarized. It is emphasized that 100% of the patients did not receive complete and correct orientations about the PAMGC and that only one professional (2.5%) referred practicing all of the following actions: requesting the capillary glucose measures to the patients for the sake of therapeutic decision making, advising on a diet, increased physical exercise and smoking cessation (when relevant) and requesting glycated hemoglobin, fasting glucose and lipid profile tests at the required intervals. The outcome indicators showed that only 9.7% of the patients were skilled to take conducts in response to the capillary glucose measures obtained at home. According to 86.4% of them, the participation in the PAMGC was positive for the sake of self-care and disease control. Among the professionals, 81.3% referred that the Program offered benefits for the purpose of disease control.

**Table 4:** - Synthesis of structure, process and outcome indicators (in percentages) for the Distribution Program of Glucometers and Inputs for Self-Monitoring of Capillary Glucose, Botucatu, SP, Brasil, 2010

Indicators		% of professional or patients
Structure		
	Professionals who know the registration criteria and disease control parameters	-
Process		
	Professionals who request capillary glucose levels during consultations, advise on diet, physical exercise and smoking cessation, when relevant, and request glycated hemoglobin, fast glucose and lipid profile, urea and creatinine tests.	2,5
	Patients who follow recommendations on number of glucose measures	15,3
	Patients with glycated hemoglobin, fasting glucose and lipid profile, urea and creatinine tests during 12 months before interview date.	18,0
Outcome		
	Professionals who consider that the PAMGC improved the patients’ disease control	81,3
	Patients with skills to act on glucose levels	9,7
	Patients who consider the inclusion in the PAMGC positive for their care and control	86,4

## Discussion

The structure, process and outcome assessment of the PAMGC in the city of Botucatu evidenced weaknesses and contradictions. The analysis of the Program structure revealed that the professionals and patients are insufficiently trained to obtain the potential benefits of glucose self-monitoring. Glucose monitoring without a plan that implies the patients' involvement with the team or the underuse of the outcomes for the sake of therapeutic adequacy do not contribute to improve the glucose control, thus indicating that monitoring alone is not effective^(^ 13 ^)^.

This shortage may affect the negative process and outcome indicators. It should be highlighted that the analysis of the quality of care delivery showed that the percentage of patients enrolled in the Program with knowledge and skills to take autonomous conducts, based on the results of the capillary glucose self-monitoring at home, was about 10%. 

Besides the deficient technical aspects related to the patients' registration in the Program and the implementation of the recommended actions, the professionals' lack of preparation can explain the lack of regular educative activities in the Program under study. It was verified that the orientations offered address only part of the strategic content the Program proposes with regard to clinical care, health promotion, management of capillary glucose self-monitoring at home and technical updates related to DM^(^ 4 ^,^ 10 ^)^. 

In view of the public policies related to the adoption of healthy life habits^(^ 14 ^)^ and their particular importance in the therapeutic plan for diabetes patients on insulin^(^ 15 ^)^, the health professionals' actions lacked expression, as almost half of them (46.9%) do not explore fundamental issues to achieve metabolic control during the consultations, such as dietary compliance, increased physical exercise and the importance of weight loss and smoking cessation. 

These results are in accordance with a study undertaken in a sample of resident physicians, which evidenced that most of them experienced difficulties to use capillary glucose self-monitoring after receiving training about DM treatment^(^ 16 ^)^. Thus, it is acknowledged that training about handling equipment and inputs should be permanent, as well as about how to interpret the results of the capillary glucose self-monitoring at home. The professionals and health service users' lack of training puts the utility of this technology at risk as a tool to reduce complications and enhance disease control in the city under study, influencing the autonomy for self-care. 

The finding that most of the physicians indicate that they do not advise the users to adjust the insulin doses according to the capillary glucose measures obtained reveals an underlying problem, which is the professionals' disbelief in the users' self-care ability to adjust the treatment with a view to their autonomy. These results can be validated by the finding that, in most cases, the professionals do not even verify the worksheets with the capillary glucose levels at home during the consultations. It should be emphasized, however, how important it is for the professionals to share the decisions about the therapeutic plan with the users, favoring the right to know and decide on their own health^(^ 17 ^-^ 18 ^)^. A study to assess the metabolic control of patients registered in the PAMGC, held in Ribeirão Preto-SP, showed that the metabolic control improved even without systematic monitoring by the multidisciplinary health team, characterized by the significant reduction of HbA1C^(^ 10 ^)^.

Based on the constructed structure, process and outcome indicators, it could be identified that one of the main objectives of the PAMGC, the joint construction of self-care skills^(^ 4 ^,^ 11 ^,^ 19 ^)^, has not been achieved in the city. Further research is needed, however, to compare the Program outcomes in different locations or regions of the country, with a view to defining their external validity. In summary, it needs to be defined whether the negative indicators are a particularity of the city under study or whether the PAMGC has weaknesses at the national level. 

The contradiction between the negative structure and process indicators and the health service users and professionals' perceived positive impact of the Program arouses questions about the utility of satisfaction as an outcome indicator. In fact, the health service users are reluctant to express criticism and dissatisfaction with the health services where they are monitored. In addition, their perceptions can be influenced by their expectations, earlier experiences or current health condition^(^ 20 ^)^. Thus, access to equipment and inputs without any counterpart, i.e. as a right, may have been sufficient for the positive assessments. It is known that, in many chronic illness situations, like DM and cancer for example, access to medication and/or inputs needed for treatment has often depended on legal proceedings^(^ 21 ^)^.

The results of this assessment strongly suggest that the local primary health care managers need to prioritize the implementation of the PAMGC. The value of the program is beyond doubt but, besides the registration of DM patients on insulin, the indication of capillary glucose self-monitoring at home and the provision of material, the health professionals need better skills in order to verify the benefits of this technology, offering training, updated knowledge, pedagogical skills for communication, listening and understanding in dealing with the health users, so as to enable them to verify the benefits of this technology^(^ 22 ^)^. 

One of the limitations of this study was the absence of the service managers responsible for putting the PAMGC in practice. In future studies, their inclusion is recommended with a view to better understanding their conceptions and actions regarding the Program.

The main hypothesis to explain the negative results obtained is related to the lack of clarity about the objectives of the PAMGC among the main actors: professionals and health service users. The professionals seemed to believe that their responsibility was to comply with the federal law that established the free distribution of glucometers and inputs, instead of the effective implementation of the PAMGC for the DM patients on insulin under their care. The users also seemed to have a limited conception about their role in the Program: simply obtaining the equipment and material for capillary glucose measuring at home. As a result, the use of capillary glucose self-monitoring at home to gain benefits from this technology remains a challenge for primary health care in the city under study.

## Conclusion

Based on the results, it can be concluded that, with regard to the structure, most of the health professionals were not appropriately trained and were not familiar with the criteria for registering the users in the Program. The process indicators showed that, in comparison with the physicians, adherence levels to official protocols were higher among the nurses. What the outcome indicators is concerned, 86.4% of the patients and 81.3% of the professionals assessed the Program positively. On the whole, through the constructed assessment indicators, it could be identified that one of the main objectives of the Program, the development of self-care skills for diabetes patients on insulin, has not been achieved.

## References

[1] Ministério da Saúde(BR) (2007). Política Nacional de Avaliação de Desempenho do SUS.

[2] Donabedian A (1991). Striving for Quality in Health Care. An Inquity into Policy and
Practice.

[3] Donabedian A (1966). Evaluating the quality of medical care. Milbank Mem Fund Q..

[4] Ministério da Saúde(BR) (2006). Lei nº.11.347. Dispõe sobre a distribuição gratuita de
medicamentos e materiais necessários à sua aplicação e à monitoração da glicemia
capilar aos portadores de diabetes inscritos em programas de educação para
diabéticos. Diário Oficial da União.

[5] American Diabetes Association (2013). Standards of medical care in diabetes. Diabetes Care.

[6] Sociedade Brasileira de Diabetes (2009). Diretrizes da Sociedades Brasileira de Diabetes.

[7] Polonsky WH, Fisher L, Schikman CH, Hinnen DA, Parkin CG, Jelsovsky Z (2011). A structured self-monitoring of blood glucose approach
in type 2 diabetes encourages more frequent, intensive, and effective physician
interventions: results from the STeP study. Diabetes Technol Ther..

[8] Poolsup N, Suksomboon N, Rattanasookchit S (2009). Meta-analysis of the benefits of self-monitoring of
blood glucose on glycemic control in type 2 diabetes patients: an
update. Diabetes Technol Ther..

[9] Grossi SA, Lottemberg SA, Lottemberg AM, Della Manna T, Kuperman H (2009). Home blood glucose monitoring in type 1 diabetes
mellitus. Rev. Latino-Am. Enfermagem..

[10] Veras VS, Araújo MFM, Rodrigues FFL, Santos MA, Damasceno MMC, Zanetti ML (2012). Assessment of metabolic control among patients in a
capillary glucose self-monitoring program. Acta Paul Enferm..

[11] Ministério da Saúde(BR) (2007). Portaria nº 2.583. Define elenco de medicamentos e
insumos disponibilizados pelo Sistema Único de Saúde, nos termos da Lei nº 11.347,
de 2006, aos usuários portadores de diabetes mellitus. Diário Oficial da União.

[12] Ministério da Saúde(BR) (2006). Cadernos de Atenção Básica nº16 - Diabetes Mellitus.

[13] Grossi SAA, Cianciarullo TI, Manna TD (2003). Caracterização dos perfis glicêmicos domiciliares como
estratégia para os ajustes insulinoterápicos em pacientes com diabetes mellitus do
tipo 1. Rev Esc Enferm USP.

[14] Ministério da Saúde(BR) Política Nacional de Promoção da Saúde. [Internet].

[15] Guzmán JR, Lyra R, Aguilar-Salinas CA, Cavalcanti S, Escano F, Tambasia M (2010). Treatment of type 2 diabetes in Latin America: a
consensus statement by the medical associations of 17 Latin American
countries. Rev Panam Salud Publica.

[16] Zanoni PH, Parisi MCR, Admoni SN, Queiroz MS, Nery M (2009). Curso de imersão em diabetes como técnica educativa para
profissionais médicos. Arq Bras Endocrinol Metabol..

[17] Teixeira CRS, Zanetti ML, Pereira MCA (2009). Perfil de diagnósticos de enfermagem em pessoas com
diabetes segundo modelo conceitual de Orem. Acta Paul Enferm..

[18] Fortes PAC (2004). Ética, direitos dos usuários e políticas de humanização
da atenção à saúde. Rev Saúde Soc..

[19] Montenegro RM Junior, Silveira MMC, Nobre IP, Silva CAB (2004). Assistência multidisciplinar e o manejo efetivo do
diabetes mellitus: desafios atuais. RBPS.

[20] Zanetti ML, Otero LM, Biaggi MV, Santos MA, Péres DS, Guimarães FPM (2007). Satisfaction of diabetes patients under follow-up in a
diabetes education program. Rev. Latino-Am. Enfermagem..

[21] Chieffi AL, Barata RB (2009). Judicialização da política pública de assistência
farmacêutica e equidade. Cad Saúde Pública.

[22] Santos MA, Peres SP, Zanetti ML, Otoro LM, Teixeira CRS (2009). Programa de educação em saúde: expectativas e benefícios
percebidos por pacientes diabéticos. Rev Enferm UERJ.

